# Effectiveness of Telemonitoring in Reducing Hospitalization and Associated Costs for Patients With Heart Failure in Finland: Nonrandomized Pre-Post Telemonitoring Study

**DOI:** 10.2196/51841

**Published:** 2024-02-07

**Authors:** Jorma Kokkonen, Pirjo Mustonen, Eija Heikkilä, Riikka-Leena Leskelä, Paula Pennanen, Kati Krühn, Arto Jalkanen, Jussi-Pekka Laakso, Jari Kempers, Sami Väisänen, Paulus Torkki

**Affiliations:** 1 Tampere Heart Hospital Tampere Finland; 2 The Wellbeing Services County of Southwest Finland Turku Finland; 3 Nordic Healthcare Group Helsinki Finland; 4 Roche Diagnostics (Schweiz) AG Zug Switzerland; 5 The Wellbeing Services County of South Savo Mikkeli Finland; 6 Ceneron Advisor Oy Helsinki Finland; 7 European Health Economics Oy Jyväskylä Finland; 8 Roche Diagnostics Oy Espoo Finland; 9 Department of Public Health Faculty of Medicine University of Helsinki Helsinki Finland

**Keywords:** cost, Finland, heart failure, hospital, resource use, telemonitoring

## Abstract

**Background:**

Many patients with chronic heart failure (HF) experience a reduced health status, leading to readmission after hospitalization despite receiving conventional care. Telemonitoring approaches aim to improve the early detection of HF decompensations and prevent readmissions. However, knowledge about the impact of telemonitoring on preventing readmissions and related costs remains scarce.

**Objective:**

This study assessed the effectiveness of adding a telemonitoring solution to the standard of care (SOC) for the prevention of hospitalization and related costs in patients with HF in Finland.

**Methods:**

We performed a nonrandomized pre-post telemonitoring study to estimate health care costs and resource use during 6 months on SOC followed by 6 months on SOC with a novel telemonitoring solution. The telemonitoring solution consisted of a digital platform for patient-reported symptoms and daily weight and blood pressure measurements, automatically generated alerts triggering phone calls with secondary care nurses, and rapid response to alerts by treating physicians. Telemonitoring solution data were linked to patient register data on primary care, secondary care, and hospitalization. The patient register of the Southern Savonia Social and Health Care Authority (Essote) was used. Eligible patients had at least 1 hospital admission within the last 12 months and self-reported New York Heart Association class II-IV from the central hospital in the Southern Savonia region.

**Results:**

Out of 50 recruited patients with HF, 43 completed the study and were included in the analysis. The hospitalization-related cost decreased (49%; *P*=.03) from €2189 (95% CI €1384-€2994; a currency exchange rate of EUR €1=US $1.10589 is applicable) during SOC to €1114 (95% CI €425-€1803) during telemonitoring. The number of patients with at least 1 hospitalization due to HF was reduced by 70% (*P*=.002) from 20 (47%) out of 43patients during SOC to 6 (14%) out of 43 patients in telemonitoring. The estimated mean total health care cost per patient was €3124 (95% CI €2212-€4036) during SOC and €2104 (95% CI €1313-€2895) during telemonitoring, resulting in a 33% reduction (*P*=.07) in costs with telemonitoring.

**Conclusions:**

The results suggest that the telemonitoring solution can reduce hospital-related costs for patients with HF with a recent hospital admission.

## Introduction

The prevalence of heart failure (HF) and related costs is increasing worldwide due to an aging population [1]. The estimated prevalence of HF in the adult population is 1% to 2%, increasing to 10% in older adults aged 70 years or older [2,3]. HF often leads to gradual or acute changes in HF symptoms (decompensation) that require repeated and prolonged hospitalization [4]. Hospital admission is a strong predictor of further hospital admission: 20% to 25% of patients with HF are rehospitalized within 1 month and approximately 50% within 5 months of discharge [5]. Decompensation requiring hospitalization is also linked to increased mortality. A European registry study following patients for 1 year after hospitalization reported mortality rates of 24% for acute HF and 6.4% for chronic HF [6]. Hospitalization accounted for around 80% of HF health care costs [1].

An early return to the hospital following discharge may be a result of incomplete inpatient treatment and poor coordination and planning of follow-up care. Even for patients with regular follow-up care, however, the signs of decompensation may not occur during cardiology visits. Patients often contact clinics when symptoms are at an advanced stage [7]. Self-monitoring of symptoms, such as increased blood pressure, weight gain, or other health status-related symptoms, is particularly important in HF management [4]. Self-monitoring requires patients to be motivated to measure symptoms associated with HF and to have access to clinical advice when symptoms appear [8].

Remote monitoring aims to improve monitoring of patients’ health status and is defined as a part of telehealth [9]. A basic level of remote monitoring involves regular and structured telephone support provided by health care professionals (HCPs) to discuss symptoms, self-monitoring measurements, lifestyle, and drug therapy. Structured telephone support can reduce HF-related hospitalization but does not seem to have an impact on the all-cause hospitalization of patients with HF [10]. Remote monitoring solutions are noninvasive stand-alone systems in which patient data on biometric measurements (such as body weight, blood pressure, and heart rate) and reported symptoms are frequently transmitted to HCPs through a secure digital system. HCPs manually review the data on digital platforms, which may also include integrated automated alerts, and necessary action is taken to optimize treatment.

The effect of noninvasive telemonitoring has been compared to the standard of care (SOC) in several studies, primarily through randomized trials. Some studies found telemonitoring had a beneficial impact on reducing hospitalization [11], while others did not find any effect [12,13]. However, a recent meta-analysis, encompassing 91 randomized trials and observational studies, revealed that noninvasive telemonitoring reduced all-cause mortality by 16%, first hospitalization by 19%, and total HF hospitalizations by 15%. When comparing telemonitoring studies and developing optimal telemonitoring approaches, it is crucial to consider various determinants, including the telemonitoring intervention models, health care systems, and the characteristics of the population with HF in the studies [14].

There are only a few international studies that have explored the cost-effectiveness of telemonitoring compared to the SOC [15-18].

The objective of this nonrandomized pre-post intervention study in patients with HF with a recent (<12 month) hospitalization was to assess the effectiveness of adding a telemonitoring solution to SOC on hospitalizations and related costs in the Finnish health care system. The study compared hospitalization occurrence and related costs with SOC and following the introduction of a telemonitoring solution. Secondary outcomes included hospital admissions and total health care costs.

## Methods

### Study Design

The nonrandomized pre-post intervention study was performed in Southern Savonia, Finland. During the 12-month study period, patients were treated with SOC for the first 6 months and then with a telemonitoring solution in addition to SOC for the next 6 months. The primary outcome was hospitalization-related costs during 6 months with SOC versus telemonitoring. Secondary outcomes included the number of patients with at least 1 hospital admission due to HF or a cardiovascular cause other than HF emergency care visits and primary care or cardiology (secondary care) calls and visits. Health care costs for secondary outcomes included the total health care costs of primary care, secondary care (for cardiology), emergency visits, and phone calls. The study was designed to demonstrate the effectiveness of remote monitoring within the Finnish health care system. The costs of the telemonitoring service itself were not analyzed.

Health care resource use was collected for each patient during SOC and telemonitoring from the patient register of the Southern Savonia Social and Health Care Authority (Essote). The data was pseudonymized by the register holder. The Health Care Authority is responsible for all social and health care services for the population of approximately 100,000 inhabitants in Southern Savonia, Finland. 

### Study Patients

**Figure 1:**
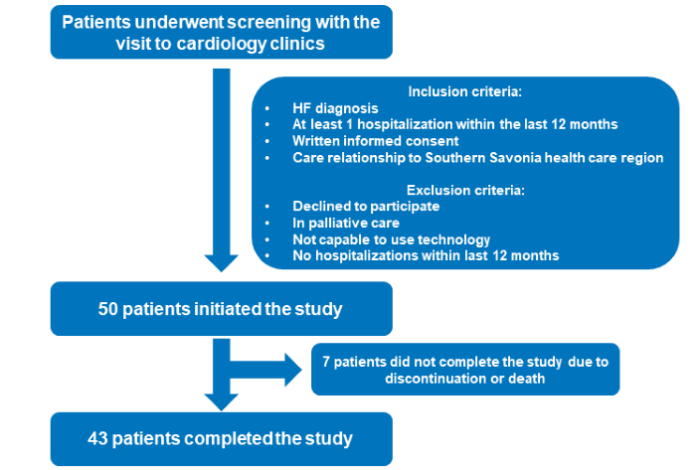
Flowchart for patient selection for the study.

Patients were recruited from Mikkeli Central Hospital in Finland’s Southern Savonia region. Patients with an HF diagnosis confirmed by a cardiologist, at least 1 hospital admission in the 12 months preceding study initiation, and self-reported New York Heart Association (NYHA) class II-IV were eligible for the study (Figure 1).

The inclusion criteria also stated that patients must be able to manage the telemonitoring devices and digital platform used in the study. Palliative care was an exclusion criterion.

### Study Procedures

The SOC, during the first 6 months, included regular cardiology appointments and laboratory tests planned by a cardiologist for each patient with HF according to local care guidelines for HF treatment. Nurses followed up with patients through phone calls, depending on the state of HF. After inpatient stays, the cardiologist or internist at the hospital made an individual plan for the follow-up of patients posthospitalization. During the follow-up period, patients measured their weight and blood pressure at home, and nurses followed up with patients through phone calls to discuss their health status and measurement results.

**Figure 2:**
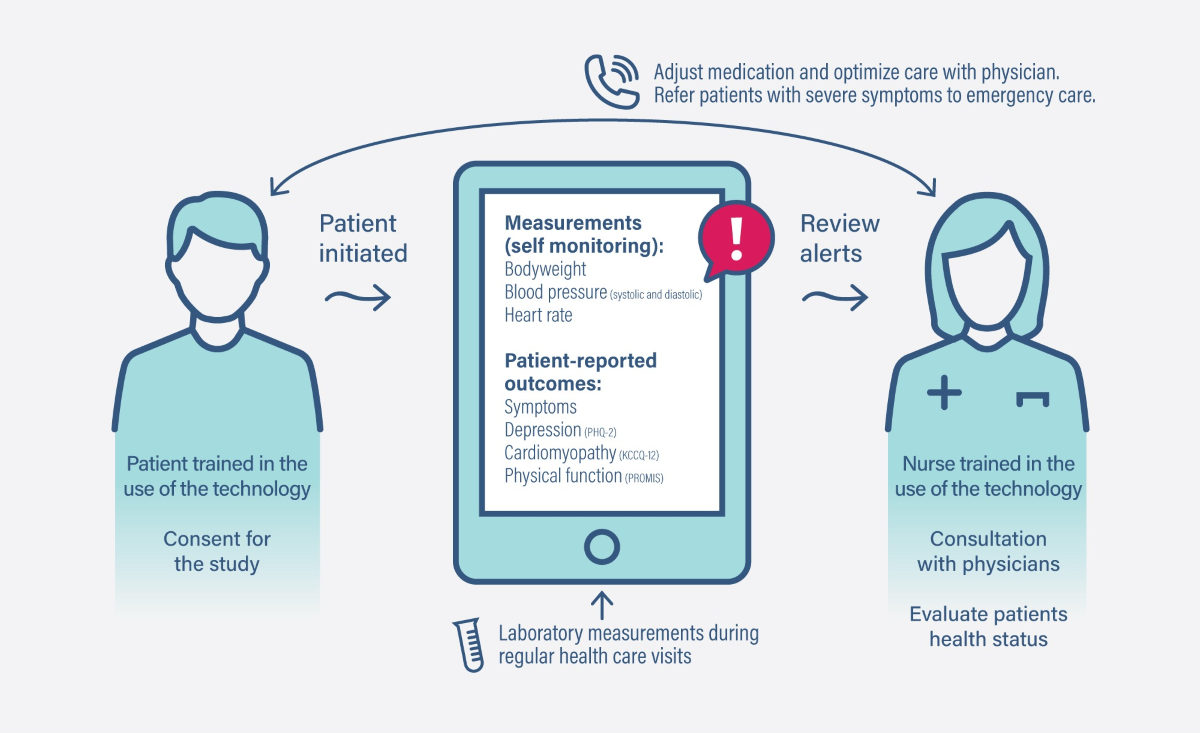
A schematic presentation of the remote patient management model. KCCQ-12: Kansas City Cardiomyopathy Questionnaire-12; PHQ2: Patient Health Questionnaire-2; PROMIS: Patient Reported Outcomes Measurement Information System.

Telemonitoring was added on top of SOC during the next 6 months and consisted of a digital platform, home measurement devices, and nurses monitoring patients through the digital platform. The digital platform used Veta Health’s remote patient monitoring platform (Veta Health Inc), customized for the study. Patients used their smartphones, handheld devices, or personal computers to access the digital platform. Patients measured their weight daily with a digital scale (Omron Corporation) and their blood pressure with a digital blood pressure measuring device (Omron M7000 Intelli IT) and transferred the measurements into the digital platform (Figure 2). The digital platform also included symptom-related questions.

The digital platform automatically compared patients’ body weight and HF symptom answers against preset thresholds and generated semiurgent or urgent alerts predicting HF worsening (Table S1 in Multimedia Appendix 1). Depending on the alert type, the digital platform either advised the patient to contact a nurse or a nurse to contact the patient to validate the health status. Nurses had access to alerts on working days. If needed, nurses referred a patient to a cardiologist to optimize HF care or medication. The treating cardiologist reacted to the nurses’ referrals within 24 hours. For urgent alerts, the digital platform advised patients to go to emergency care. Nurses also provided technical support for patients as required. The digital platform collected blood pressure data and laboratory results from regular health care visits, not for the alert algorithm but to allow nurses to evaluate the patient’s health status.

### Health Care Resource Use and Costs

Patients’ health care costs and resource use were estimated from the Essote patient register and consisted of public primary care, secondary care (cardiology unit), emergency visits, hospitalizations (cardiology and internal medicine; primary care), and phone calls for primary and secondary care (cardiology). A unique personal identification number for each resident in Finland connected the digital platform data and patient register data. The *International Classification of Diseases, Tenth Revision* (ICD-10) diagnosis code registered for each health care event was used to separate hospitalizations for HF (ICD-10 code I50) from hospitalizations for a cardiovascular cause other than HF (ICD-10 codes I10, I25, I42, I46, I48, I49, and I70). All-cause hospitalizations included hospitalizations with any diagnosis. The costs of health care use were calculated using Essote diagnosis-related group prices.

### Statistical Analysis

The study analysis included only patients who completed the study. Patients who died during the study or discontinued the study were excluded.

Patient demographics and NYHA class were summarized as n (%) of patients per category or median (IQR). During the SOC and telemonitoring periods, n (%) of patients with at least 1 hospitalization and the mean number of inpatient days per patient (95% CI) were reported. The mean number (95% CI) of visits per patient (primary, secondary, and emergency) and the mean number of calls (primary and secondary care) per patient were also reported for each period. Mean health care costs per patient were reported for each period. The normal distribution of each variable was assessed through visual inspection and the Shapiro-Wilk test. For data found to be nonnormally distributed, differences between SOC and telemonitoring periods were tested using the Wilcoxon signed rank test, and a value of *P*<.05 was considered statistically significant. The Pearson chi-square test with Yates correction was used for testing the difference between SOC and telemonitoring periods (a binary variable) in the number of patients with at least 1 hospitalization.

### Ethical Considerations

The ethics committee of the Northern Savonia Hospital District approved the study protocol (1401/2020). The study followed good clinical practice following the Declaration of Helsinki and the laws and regulations applicable in Finland. Patients gave written consent upon recruitment to the study. Participation in the study was voluntary and no financial compensation was awarded for participation.

## Results

### Study Population and Patient Characteristics

Between December 15, 2020, and March 24, 2021, a total of 50 patients with HF were recruited from the Mikkeli Central Hospital. A total of 7 patients did not complete the study due to their deaths or withdrawals from it. All 43 (86%) patients who completed the 12-month study period were included in the analysis. During the telemonitoring period, 20% (9/43) of the daily weight and blood pressure measurements were missing.

The median age of patients was 73 (IQR 66-80) years, 74% (37/50) were male, and 60% (30/50) of patients had NYHA classes III-IV (Table 1).

**Table 1:** Patient demographics and disease characteristics (n=50).

Characteristic			Value
Age (years), median (IQR)			73 (66-80)
**Sex, n (%)**			
	Male		37 (74)
	Female		13 (26)
**NYHA^a^ score, n (%)**			
		NYHA class II	20 (40)
		NYHA class III-IV	30 (60)
Systolic blood pressure (mm Hg), mean (SD)			118 (18)
Heart rate (beats/min), mean (SD)			72 (11)
BMI (kg/m^2^), mean (SD)			27 (6)
Serum creatinine (µmol/L), mean (SD)			125 (44)
Pro–B-type natriuretic peptide (ng/L), median (IQR)			3122 (1590-5598)
Left ventricular ejection fraction (%), mean (SD)			37 (11)
**Etiology of heart failure, n (%)**			
	Ischemic cardiomyopathy		15 (30)
	Idiopathic dilated cardiomyopathy		13 (26)
	Hypertensive cardiomyopathy		12 (24)
	Tachycardia cardiomyopathy		<5
	Cytostatic cardiomyopathy		<5
	Valvular cardiomyopathy		<5
	Genetic cardiomyopathy		<5
**Medical history, n (%)**			
	Hypertension		24 (48)
	Diabetes		14 (28)
	Coronary heart disease		17 (34)
	Myocardial infarction		6 (12)
	Atrial fibrillation		31 (62)
	Valvular heart disease		<5
**Medication at recruitment, n (%)**			
	Diuretic		46 (92)
	Digitalis		6 (12)
	β-blocker		48 (96)
	Mineralocorticoid antagonist		35 (70)
	ACE^b^-inhibitor		7 (14)
	Angiotensin receptor-blocker		10 (20)
	Valsartan-sacubitril		30 (60)
	SGLT2^c^-inhibitor		<5
	Statin		26 (52)
	Anticoagulant		42 (84)
	ASA^d^ or clopidogrel		11 (22)

^a^NYHA: New York Heart Association.

^b^ACE: angiotensin-converting enzyme.

^c^SGLT2: sodium-glucose cotransporter-2 .

^d^ASA: acetylsalicylic acid.

### Health Care Resource Use

Significantly fewer patients (6 patients in telemonitoring vs 20 patients in SOC; *P*=.002) had an HF hospitalization during the telemonitoring versus SOC period. The number of inpatient days per patient due to HF decreased by 48% during the telemonitoring period (mean 1.2, 95% CI 0.1-2.3 days vs 2.3, 95% CI 1-3.6 days with SOC; *P*=.17). The number of emergency care visits decreased significantly during the telemonitoring period by 44% (mean 0.7, 95% CI 0.4-1 vs mean 1.3, 95% CI 0.9-1.7 with SOC; *P*=.006). Patients with HF made significantly more phone calls to secondary care during the telemonitoring period (mean 8.3, 95% CI 6.6-10 vs mean 2, 95% CI 1.3-2.7 with SOC; 318% increase; *P*.001) and had significantly more primary care visits (mean 4, 95% CI 2.2-5.8 vs mean 2.8, 95% CI 1.7-3.9; 44% increase; *P*=.02; Table 2).

**Table 2:** Use of health care per patient in standard of care (SOC) or telemonitoring solution for a 6-month period. Statistics were calculated with the Wilcoxon signed rank test or the Pearson chi-square test with Yates correction for the binary variables.

Variable			SOC (n=43)		Telemonitoring (n=43)		Absolute change (relative change; %)		*P* value for difference
**All-cause hospitalizations**									
	Patients with ≥1 event, n (%)	23 (53)		14 (33)		–9 (–39)		.08	
	Inpatient days, mean (95% CI)	2.9 (1.6)		1.7 (1.2)		–1.2 (–41)		.20	
**Hospitalizations for cardiovascular cause other than HF^a^**									
	Patients with ≥1 event, n (%)	6 (14)		<5 (~10)		<5 (~–30)		.70	
	Inpatient days, mean (95% CI)	0.2 (0.3)		0.02 (0.05)		–0.16 (–88)		.40	
**Hospitalizations for HF**									
	Patients with ≥1 event, n (%)	20 (47)		6 (14)		–14 (–70)		.002	
	Mean inpatient days, days (95% CI)	2.3 (1.3)		1.2 (1.1)		–1.1 (–48)		.17	
Mean number of emergency care visit for cardiovascular cause, n (95% CI)			1.3 (0.4)		0.7 (0.3)		–0.6 (–44)		.006
Mean number of phone calls to primary care, n (95% CI)			3.9 (1.3)		2.5 (0.9)		–1.4 (–36)		.01
Mean number of phone calls to secondary care, n (95% CI)			2 (0.7)		8.3 (1.7)		6.3 (+318)		<.001
Mean number of all-cause primary care visits, n (95% CI)			2.8 (1.1)		4 (1.8)		1.2 (+44)		.02
Mean number of secondary care visits (cardiology), n (95% CI)			1.8 (0.5)		1.9 (0.6)		0.1 (+8)		.80

^a^HF: heart failure.

### Health Care Costs

Mean hospitalization costs per patient decreased significantly by 49% during the telemonitoring period (mean €1114 vs €2189 with SOC; *P*=.03; a currency exchange rate of EUR €1=US $1.10589 is applicable), while total health care costs decreased by 33% (mean €2104 vs €3124 with SOC; *P*=.07; Table 3).

The cost of emergency care visits was also significantly lower in the telemonitoring period (mean €209 vs €347 with SOC; 40% decrease; *P*=.009), and mean costs per patient for phone calls to secondary care increased significantly (mean €268 vs €114 with SOC; 134% increase; *P*.001) in the telemonitoring period (Table 3).

**Table 3:** Estimated mean direct health care cost per patient in standard of care (SOC) and in telemonitoring solution, respectively, during a 6-month period (2021). A currency exchange rate of EUR €1=US $1.10589 is applicable. Statistics were calculated with the Wilcoxon signed rank test.

Cost category	SOC (n=43), mean cost (€; 95% CI)	Telemonitoring (n=43), mean cost (€; 95% CI)	Absolute change (€; relative change in mean cost; %)	*P* value for difference
Hospitalizations^a^	2189 (805)	1114 (689)	–1075 (–49)	.03
Primary care visits	75 (37)	101 (74)	27 (+36)	.30
Secondary care visits (cardiology)	288 (77)	337 (87)	50 (+17)	.20
Emergency care visits	347 (122)	209 (99)	–137 (–40)	.009
Phone calls to primary care	112 (43)	74 (35)	–38 (–34)	.02
Phone calls to secondary care	114 (42)	268 (68)	153 (+134)	<.001
Total cost	3124 (912)	2104 (791)	–1020 (–33)	.07

^a^Hospitalization from cardiology and internal medicine ward and from primary care.

## Discussion

### Principal Findings

In this pre-post study of a novel telemonitoring solution for patients with HF in Finland, hospitalization costs were significantly lower during the 6-month telemonitoring period versus the SOC period (from €2189 per patient during SOC to €1114 during telemonitoring). The number of hospitalized patients was significantly lower during telemonitoring (from 20 during SOC to 6 patients), and the mean length of stay decreased from 2.3 days to 1.2 days (not statistically significant). The number of emergency visits and associated costs were also significantly lower during telemonitoring. By contrast, patients with HF had significantly more primary care visits and phone calls to secondary care nurses during telemonitoring versus SOC; however, total health care costs were 33% lower than during SOC (not statistically significant).

The reduction in inpatient days due to HF during telemonitoring was not statistically significant, most probably due to the low number of patients with HF in the study. While secondary care phone calls increased significantly, a substantial part of these were for technical help in using the digital platform at the start of the telemonitoring period. The number of primary care phone calls, on the other hand, decreased during the telemonitoring period, which may be because secondary care nurses were monitoring patients.

### Comparison With Previous Work

A comprehensive meta-analysis of recently published studies on telemonitoring has provided evidence that telemonitoring is beneficial in reducing mortality and hospitalizations in patients with HF. However, individual studies show both beneficial and neutral effects of telemonitoring when compared to SOC [11,13,19-21]. The main objective of the studies was to investigate whether remote monitoring can improve the detection of early signs of decompensation and decrease hospitalization and mortality. The variability of the results may be due to differences in the health care system, telemonitoring model, population with HF, and follow-up durations [9,14].

This study found that 47% (20/43) of patients were hospitalized due to HF during the SOC period, versus 14% (6/43) during the telemonitoring period in the Southern Savonia region of Finland. Vuorinen et al [21] conducted a telemonitoring study in Finland from 2010 to 2012. They showed that only 28% of patients were hospitalized in the SOC group and 17% in the remote monitoring group during a 6-month follow-up period. The inclusion criteria for the population with HF included NYHA II-IV but did not require a recent hospitalization, which could explain the lower HF hospitalization risk compared with this study. Our data align with previous studies showing that nearly half of patients with HF are rehospitalized within 6 months after discharge [21,22]. However, further studies using a similar population with HF are needed to confirm these findings.

Similar to this study, Vuorinen et al [21] also found a nonstatistically significant decrease in inpatient days with telemonitoring (mean 0.7 vs 1.4 days with SOC). The significant reduction in hospitalization-related costs and the number of patients hospitalized due to HF in this study support the idea that telemonitoring reduces hospitalizations.

A Spanish telemonitoring trial (n=117) had similar findings to this study. In this trial, 50% of the patients were hospitalized in the SOC group versus 28% in the telemonitoring group over a 6-month follow-up period. The patients were enrolled in the study upon hospitalization [19]. Thus, the results from this study support our findings on the benefits of telemonitoring in reducing hospitalizations. However, a large Better Effectiveness After Transition–Heart Failure (BEAT-HF) trial (n=1437) conducted in California could not see a reduction in readmissions in patients with HF in a telemonitoring group compared to SOC during a 6-month follow-up period [20]. The BEAT-HF trial’s limitations were that patients were recruited from academic medical centers, which may restrict the generalizability of the results, as most patients with HF do not receive care in academic medical centers. Upon receiving alerts, nurses advised patients to contact the physicians, or nurses called the physicians, but physicians were not directly involved with the interventions. Thus, the monitoring may not have affected care in practice. In this study and the Spanish study, nurses and treating physicians collaborated upon receiving alerts, which may have increased the benefits of telemonitoring. For example, in this study, physicians reacted to patient alerts within 24 hours.

There are several studies on the effect of telemonitoring on hospitalization and mortality, but only a few studies on costs. This study estimated the health care cost related to resource use using real-word data and showed that health care costs were 33% (€2104/€3124) lower during telemonitoring versus SOC. As expected, most of the cost reductions originated from reduced hospitalizations. A cost-effectiveness study in a Danish telemonitoring trial (n=274) reported similar results by showing that telemonitoring reduced total health care costs by 35% versus SOC with a 1-year follow-up [16]. In a Spanish telemonitoring trial, Comin-Colet et al [15] found a total cost reduction of 45% with telemonitoring versus SOC, with 178 patients and a 6-month follow-up. A Belgian Telemonitoring in the Management of Heart Failure (TEMA-HF) study (n=160) found a 27% cost reduction (not statistically significant) with telemonitoring versus SOC during a 6-month follow-up [23]. The German Heart Failure II trial (TIM-HF2) showed an 18% reduction in annual costs per patient in the telemonitoring group compared to the SOC group during a 1-year follow-up [18]. These studies support our conclusion that telemonitoring may result in substantial cost savings in HF care. To justify reimbursement for telemonitoring, studies are needed on the cost-effectiveness of large-scale telemonitoring for decision makers. Furthermore, a cost-effective telemonitoring model applicable to different health care systems and settings needs to be developed.

### Strengths and Limitations

A strength of this study was the cost analysis, which included both HF-related health care costs as well as other costs accrued during the follow-up period. The study had some limitations. The study was conducted in a single region, the Southern Savonia region, which may limit the generalizability of the results. However, the study population is representative of the region, as all patients are directed to the same central hospital where recruitment was done. A randomized controlled trial design was not feasible due to the limited number of suitable patients with HF for remote monitoring. No patients with HF were included from other health care districts, as divergent monitoring practices could potentially bias the analysis results. Patients were not randomized, and patients needed to be able to use the digital platform, which may have resulted in a possible selection bias. As the pre-post design uses a historical control group (ie, patients on SOC in the period before starting telemonitoring), the underlying assumption in the analysis, given the deteriorating nature of HF, is that health care use in the absence of telemonitoring would remain at least at the same level as during SOC. Follow-up with telemonitoring was limited to 6 months, and it is unclear how use of health care services would develop beyond this period. Finally, due to the small patient numbers, the absence of a control group, and the 6-month follow-up period, it was not feasible to conduct mortality analyses.

The following must be considered when generalizing our results and applying our telemonitoring solution to other health care systems: our telemonitoring solution was applied to a patient population with a high risk of readmission due to a recent hospital admission and NYHA class II-IV. Other patient characteristics considered were the mean age (73 years), male proportion (37/50, 74%), proportion of patients in NYHA class II-IV (30/50, 60%), and proportion of patients with at least 1 admission within 6 months (20/50, 47%). These patient characteristics were comparable to those of other reported telemonitoring study populations [11,13,18,19].

### Conclusions

In conclusion, our results suggest that the novel telemonitoring solution can help reduce hospital admissions and hospitalization costs as well as total health care costs in a population with HF with a recent hospital admission in the past 12 months.

## Appendix

Multimedia Appendix 1 Definition of the alert triggers.

## Abbreviations

BEAT-HF: Better Effectiveness After Transition–Heart Failure
HCP: health care professional
HF: heart failure
ICD-10: International Classification of Diseases, Tenth Revision
NYHA: New York Heart Association
SOC: standard of care
TEMA-HF: Telemonitoring in the Management of Heart Failure
TIM-HF2: German Heart Failure II trial
