# Supplementary material for: Effectiveness of Telemonitoring in Reducing Hospitalization and Associated Costs for Patients With Heart Failure in Finland: Nonrandomized Pre-Post Telemonitoring Study
Source: JMIR Mhealth Uhealth. 2024 Feb 7;12:e51841. doi: 10.2196/51841 (PMC10896481; doi:10.2196/51841)
Supplement: Multimedia Appendix 1 [file mhealth_v12i1e51841_app1.docx]

**Table S1.** Definition of the alert triggers

| Alert type | Alert triggers | Actions of healthcare professionals |
| --- | --- | --- |
| Semi-urgent | Weight | Optimize diuretic treatment upon need |
| Semi-urgent | HF-related symptom query, PHQ-2, KCCQ-12, Promis-4A | Nurse contacts a patient on the following day if symptoms and queries indicate that a patient has difficulties surviving in daily life based on biweekly questionnaires |
| Semi-urgent | Symptoms such as arrhythmia, fatigue, edema | Patient contact nurse on the following day latest, and the treatment is optimized upon need |
| Urgent | Symptoms such as chest pain, shortness of breath | Patient is advised urgently to contact emergency care |
| Used as background information when evaluating a patient’s health status | Laboratory values   - Creatinine - Natrium - Kalium - NT-proBNP - Hemoglobin - Hematocrit | Optimize treatment upon need |
| Used as background information when evaluating a patient’s health status | Blood pressure | Optimize treatment upon need |
